# Functionalization of Single-Walled Carbon Nanotubes with End-Capped Polystyrene via a Single-Step Diels–Alder Cycloaddition

**DOI:** 10.3390/polym13071169

**Published:** 2021-04-06

**Authors:** Maria-Malvina Stathouraki, Christos Pantazidis, Emmanouil Mygiakis, Apostolos Avgeropoulos, Georgios Sakellariou

**Affiliations:** 1 Department of Chemistry, National and Kapodistrian University of Athens, Panepistimiopolis Zografou, 15771 Athens, Greece; marmalsta@chem.uoa.gr (M.-M.S.); christospanta.chem@gmail.com (C.P.); manolis-myg@chem.uoa.gr (E.M.); 2 Department of Materials Science and Engineering, University of Ioannina, 45110 Ioannina, Greece; aavger@uoi.gr

**Keywords:** single-walled carbon nanotubes, [4+2] Diels–Alder cycloaddition, surface functionalization, anionic polymerization

## Abstract

A facile, single-step, [4+2] Diels–Alder cycloaddition reaction for the surface functionalization of single-walled carbon nanotubes (SWNTs) with end-capped polystyrene chains is presented. The thermal cycloaddition reaction took place at high temperature (~230 °C) without any catalyst between the *sp*^2^ network of carbon nanotubes, which acted as dienophile, and the diphenylethylene cyclobutene (DPE-CB) terminal group of the polystyrene chain. Anionic polymerization was employed for the synthesis of the polystyrene macromolecule, and successful and quantitative end-capping reaction with the DPE-CB molecule was confirmed by matrix-assisted laser desorption/ionization time of flight mass spectroscopy. Thermogravimetric analysis revealed the wt % of the grafted macromolecule on the CNT surface as well as the grafting density of the polymer chains on the SWNTs (0.027 chains nm^−2^). Direct evidence for the surface functionalization and the presence of thin polystyrene film was obtained by transmission electron microscopy (TEM) and by atomic force microscopy (AFM).

## 1. Introduction

Carbon nanotubes (CNTs) are promising nanomaterials due to their extraordinary electronic, and mechanic properties [1,2]. The combination of their high surface area with the strong van der Waals attractive interactions, due to the hexagonal *sp*^2^ carbon network, forces CNTs to create aggregated bundles. To realize their potential, efficient and homogeneous dispersion of CNTs in solvents and polymers is required. Homogeneous dispersion of carbon nanotubes in polymer matrices is important to develop ultrahigh strength nanocomposites that would have an efficient load transfer and a good interfacial adhesion between the CNTs and the polymer [3,4,5]. In general, debundling of carbon nanotubes is accomplished through functionalization of CNTs using organic compounds. Over the past few years, great effort has been devoted to covalent and non-covalent functionalization of CNTs. Small molecules as well as macromolecules have been employed for this purpose using a rich variety of different strategies.

The non-covalent polymer functionalization strategy generally involves the interaction of electron rich macromolecules with π–π interactions of carbon nanotubes. Ionic interactions as well as CH–π or OH–π interactions are also possible [6]. On the other hand, covalent grafting of polymers involves two different strategies, “grafting to” [7,8,9,10] and “grafting from” or surface-initiated polymerization methodology [11,12,13]. Several surface covalent functionalization strategies also involve direct addition reaction to the *sp*^2^ carbon of the CNTs with (i) nitrenes [14], (ii) diazonium salts [15], (iii) carbenes [16], (iv) azomethine ylides [17], (v) free radicals [18], (vi) fluorine [19], (vii) 1,3 dipolar cycloaddition [20], (viii) bromomalonates [21], and oxidation reactions [22,23]. All the above methodologies require demanding reaction conditions, and multiple steps are necessary to functionalize the carbon nanotubes.

Experimental [24,25,26,27,28,29,30,31] as well as theoretical studies [32] in recent years have shown that the Diels–Alder (DA) reaction is a powerful tool for the covalent functionalization of carbon nanotubes [33]. From the theoretical point of view [32], it has been shown that DA reaction can occur between the dienenophile CNTs and conjugated dienes. Specifically, it was predicted that the DA cycloaddition of *ο*-quinodimethane on the sidewalls of SWNTs was favored due to the aromatic stabilization of the transition state and the product. Our group studied the reaction of several substituted benzocyclobutenes with the *sp*^2^ network of multi-walled carbon nanotubes [34]. We reported that the extent of functionalization can be controlled by the temperature, the mode of addition of the benzocyclobutene derivative, the reaction time, and whether the reaction takes place in bulk vs. solution. A number of initiators based on benzocyclobutene were synthesized by our group and used for the surface-initiated polymerizations after the initiators were covalently attached on the CNT surface through the Diels–Alder thermal cycloaddition [35,36,37,38,39,40]. Benzocyclobutene derivatives were also used for the intramolecular [41,42] and the intermolecular crosslinking reactions for the synthesis of polymer nanoparticles [43].

Herein, we report a facile, single-step covalent strategy for the surface functionalization of SWNTs. A well-defined polystyrene chain with a diphenylethylene cyclobutene end-group was synthesized by anionic polymerization and grafted to the side CNT walls through a thermal [4+2] Diels–Alder cycloaddition reaction in the absence of any catalyst. The structural integrity of the SWNTs was retained, and the reaction did not require any surface pre-modification of the CNTs.

## 2. Materials and Methods

### 2.1. Materials

All chemicals were purchased from Sigma Aldrich, Athens, Greece.. The SWNTs (length 1 μm to 3 μm and diameter 1–1.2 nm, 93% purity, Carbon Nanotechnologies, TX) were prepared through the HiPco method and purified by multistage oxidation in moisture air at 225, 325, and 425 °C for 2 h, as reported elsewhere [44]. Single-walled carbon nanotubes (SWCNTs) had approximately 1 mol.% carboxylic acid groups with respect to the carbon atoms of the SWNTs, as determined by titration, X-ray photoelectron spectroscopy (XPS), and prompt-gamma methods. SWNTs were vacuum dried in the oven at 200 °C overnight before being used for surface functionalization. All solvents and monomers were purified according to well-known procedures [45,46]. Dimethylformamide (DMF) was distilled over NaOH. Other solvents such as ethyl acetate, diethyl ether, and hexane were used as received. The 4-bromobenzocyclobutene was distilled under vacuum and stored under N_2_. *sec*-butyllithium (*s*-BuLi), synthesized by the reaction of *sec*-butyl chloride and lithium metal, was used as the polymerization initiator. The concentration of *s*-BuLi was determined using standard styrene polymerization under a high vacuum and the measured number average molecular weight of the obtained polystyrene at 100% conversion. The detailed synthesis of diphenylethylene cyclobutene (DPE-CB) is reported elsewhere [30], and ^1^H NMR spectrum is in the supporting information (Figure S1).

### 2.2. Physical and Analytical Methods

Size exclusion chromatography (SEC) was used to determine molecular weights and molecular weight distributions (*M_w_*/*M_n_*) of the polymer samples with respect to polystyrene standards. The unit was equipped with an isotactic pump (Knauer K-501), UV detector (Knauer UV-K2501), and RI detector (Knauer RI-K2301). Polymer samples were analyzed using Polymer Standard Service (PSS, Germany) 1 × 100 Å and 1 × liner SDV 5 μm gel columns (60 cm) set or with Polymer Laboratories 2 × mixed-BSDV gel columns (30 cm). THF was used as the mobile phase at a flow rate of 1 mL min^−1^ at 30 °C. PSS WinGPC software was used to acquire and analyze the chromatograms. In some cases, SEC was performed on an SEC-LS system consisting of an RI detector (Polymer Laboratories), light scattering 15° and 90° (Precision Detectors, λ = 685 nm, 30 Mw), and viscosity (Viscotec) detectors. ^1^H-NMR (400 MHz) spectrum was recorded on a Brucker AC-400 spectrometer.

Matrix-assisted laser desorption-ionization mass spectra (MALDI-TOF MS) were recorded on a Bruker Autoflex II mass spectrometer (Bruker Daltonics, Billerica, MA, USA) equipped with a nitrogen laser operated at 337 nm, a frequency of 25 Hz, and an acceleration voltage of 20 kV. Trans-2-[3-(4-tert-Butylphenyl)-2-methyl-2-propenylidene]malononitrile (DCTB) (>99%, Fluka) was used as the matrix and silver trifluoroacetate (AgTFA) as the cationizing agent. Solutions of DCTB (20 mg/mL), the analyte (10 mg/mL), and the AgTFA (10 mg/mL) were prepared in THF and then mixed in a 10:2:1 ratio. A volume of 0.5 μL was applied to the target via the dried droplet method. Mass spectra were collected in reflectron mode, and the instrument was externally calibrated with polystyrene standards.

Thermogravimetric analysis was performed using a TA Q50 analyzer in air and N_2_ atmosphere at a heating rate of 10 °C/min.

FT-IR spectra were recorded using a Bio-Red Win-IR Pro instrument with a resolution of 2 cm^−1^. KBr was used to prepare sample pellets.

Raman spectra were measured with a Renishaw Invia Raman spectrometer equipped with an integral microscope (Leica DM 2500 M) and a Peltier cooled charged-coupled detector. The 785 (or 514) nm radiation from a 500 mW diode laser was used as excitation source, operated at 25 mW in order to avoid thermal effects. The excitation was performed through a 50× objective lens, and Raman scattering was detected in a backscattering geometry (180°). The spectral resolution using an 1800 grooves/mm grating was approximately 2 cm^−1^. Spectra were recorded over the range of 100–2000 cm^−1^ for 10–12 acquisitions (with 20 s/acquisition) and averaged. The spectra were smoothed with a 7 points adjacent averaging smooth function.

Atomic force microscopy (AFM) images were acquired in air, under ambient conditions, using an Asylum Research MFP-3D™ microscope operated in the tapping (non-contact) mode. Silicon probes (Olympus) with tip radius of 10 nm, spring constant of 42 N/m, and resonance frequency 300 kHz were used for imaging. The scan rate was varied between 0.6 and 1 Hz. The scan lines and the scan points were varied between 256 and 512. Typical sample preparation for the AFM imaging involved dispersion of carbon nanotubes in chloroform (at concentrations of 0.5 mg CNTs or less per ml of solvent), spin-coating on silicon substrates, and subsequent drying for 2 h at ambient conditions. The diameters of pristine or grafted CNTs were determined using AFM height measurements and section analysis.

Transmission electron microscopy (TEM) samples were prepared by dropping an ~5 μL solution (SWNTs-*g*-PS in chloroform) on a freshly glow discharged carbon film supported by a 600 mesh copper grid (Electron Microscopy Sciences, Hatfield, PA, USA). A JEOL 200CX electron microscope (JEOL Ltd., Tokyo, Japan), operated at 200 kV in the bright field mode, was used to examine the samples.

### 2.3. Synthesis of Diphenylethylene-Cyclobutene End-Capped Polystyrene, 1.

**Scheme 1:**
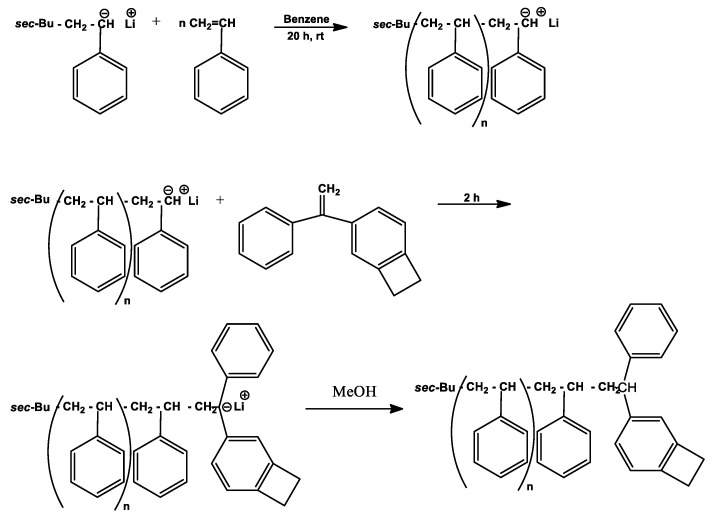

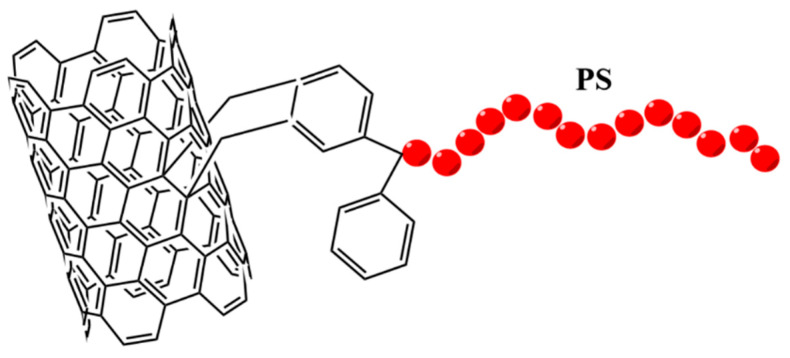
Synthetic strategy for the synthesis of end-capped PS-(diphenylethylene cyclobutene (DPE-CB)) and the surface functionalization of single-walled carbon nanotubes (SWNTs) with the DPE-CB end-capped polystyrene, 1, through a [4+2] Diels–Alder thermal cycloaddition.

**Figure 1:**
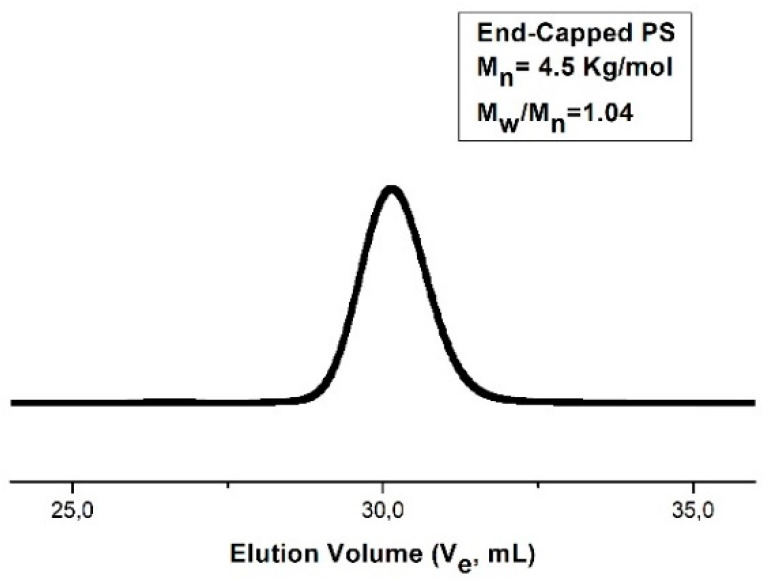
Size exclusion chromatography eluogram of DPE-CB end-capped PS 4.5 Kg/mol.

The anionic polymerization of styrene (S) was performed in benzene using standard all-glass high-vacuum techniques, Scheme 1. In a typical reaction, the required amount of S (4 g) was mixed with known amount of *s*-BuLi (9.3 × 10^−4^ mol) in benzene at room temperature under high vacuum. Immediately, an orange color developed, indicating the formation of the propagating anion of PS^−^Li^+^. The polymerization was allowed to proceed for 20 h at 25 °C. A small aliquot was taken and terminated with methanol for molecular weight characterization before the introduction of an appropriate amount of DPE-CB (3 times excess compared to the living PS-Li^+^, 2.8 × 10^−3^ mol) for the end-capping reaction (with or without THF). Immediately after mixing of the excess DPE-CB with the “living” PS^−^Li^+^, the solution color changed to dark red. The reaction was allowed to proceed for a few hours at 25 °C and was terminated with methanol (0.5 mL) under vacuum. The reactor was opened, and the polymer was recovered by precipitation in excess methanol. The end-capped polystyrene was dried under vacuum at 40 °C for 24 h. The yield was 96% (3.85 g). As shown in Figure 1, all samples exhibited narrow molecular weight distributions.

### 2.4. Synthesis of SWNTs-g-PS

In a typical experiment, 20 mg of pristine SWNTs were dispersed in 10 mL of dibenzyl ether in a glass tube and placed in an ultrasonic bath. After dispersing the solution, the glass tube was introduced in a silicone oil bath at ~230 °C with vigorous stirring under nitrogen atmosphere and left to thermostat for 10 min. A polymer solution, 500 mg in 10 mL of benzyl ether, was added drop-wise into the hot dispersion of SWNTs using a peristaltic pump at a rate of 10 mL/h. After the addition, the mixture was left for 40 more min at the oil bath. In order to be sure that the polystyrene was covalently grafted and not physisorbed on the surface of CNTs, the polymer/CNTs solution after the completion of the grafting reaction was filtered through a Teflon membrane (0.2 μm pore) and washed with copious amount of THF. The washing was repeated (three times/500 mL each time) until the filtrate showed no weight change by thermogravimetric analysis (Scheme 1)

## 3. Results and Discussion

The end-capping reaction of “living” PS^−^Li^+^ with the three-fold mol excess of PDE-CB molecule took place in a benzene solution with and without (~1 mL) THF. The orange color of the “living” PS^−^Li^+^ solution changed instantaneously upon the addition of the excess of DPE-CB to dark red, which was initial evidence of the successful reaction. This reaction was previously thoroughly studied by our group, where seven DPE-CB molecules were introduced at precise positions along a polystyrene chain [47]. The size exclusion chromatography eluogram revealed a monomodal, symmetric, low molecular weight distribution peak after the end-capping reaction without any byproducts.

**Figure 2:**
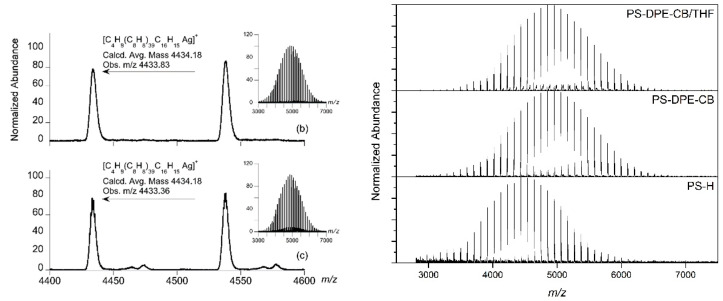
Matrix-assisted laser desorption-ionization mass spectra (MALDI-TOF MS) spectra of the PS-DPE-CB, and the PS-DPE-CB/THF.

The quantitative functionalization of PSLi with DPE-CB was confirmed by MALDI-TOF MS, as shown in Figure 2. The MALDI-TOF MS spectrum of the PS sample before the reaction with the DPE-CB revealed a single distribution, which was attributed to unfunctionalized PS (PS-H) where the observed mass of *m*/*z* 4228.1 was in good agreement with the calculated average mass of 4228.1 Da for [C_4_H_9_(C_8_H_8_)_39_H·Ag]^+^. After the end-capping reaction with DPE-CB, the molecular weight of the product shifted to higher molecular weight, which was consistent with the addition of DPE-CB unit (calculated average mass 207.3 Da). In the case of PS-(DPE-CB), one distribution was observed, and the peak observed at 4433.8 was in good agreement with the calculated average mass of 4434.2 Da for [C_4_H_9_(C_8_H_8_)_39_C_16_H_15_·Ag]^+^. The same shift was observed when PSLi reacted with PS-DPE-CB in the presence of THF. For each sample, the difference between the peaks corresponded to the mass of one styrene repeat unit.

**Figure 3:**
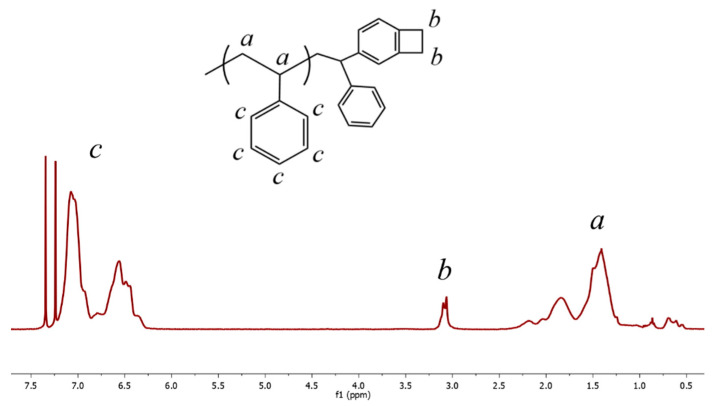
^1^H-NMR spectrum of DPE-CB end-capped PS.

The ^1^H NMR spectrum of the DPE-CB end-capped polystyrene in Figure 3 revealed the absence of vinyl protons (5.2–5.8 ppm) and the presence of aliphatic (1.0–2.2 ppm), aromatic (6.2–7.4 ppm), and cyclobutene protons (3.0–3.2 ppm), which was additional evidence for the successful end-capping reaction of the “living” PS chains with the DPE-CB molecule.

The chemical functionalization of the SWNTs with the DPE-CB end-capped PS took place in a solution of dibenzyl ether, a good solvent for PS with high boiling temperature, which is critical for the [4+2] Diels–Alder thermal cycloaddition reaction between the dienophile SWNTs and the benzocyclobutene derivative. Benzocyclobutene is a category of dienes that can take part in a Diels–Alder reaction with the *sp*^2^ carbons of an aromatic system through an *o*-quinodimethane intermediate [48]. The strained ring of the benzocyclobutene is susceptible to thermal ring opening to form *o*-quinodimethane, which can undergo [4+2] Diels–Alder cycloaddition exclusively to 6–6 bonds of fused aromatic rings [48].

**Figure 4:**
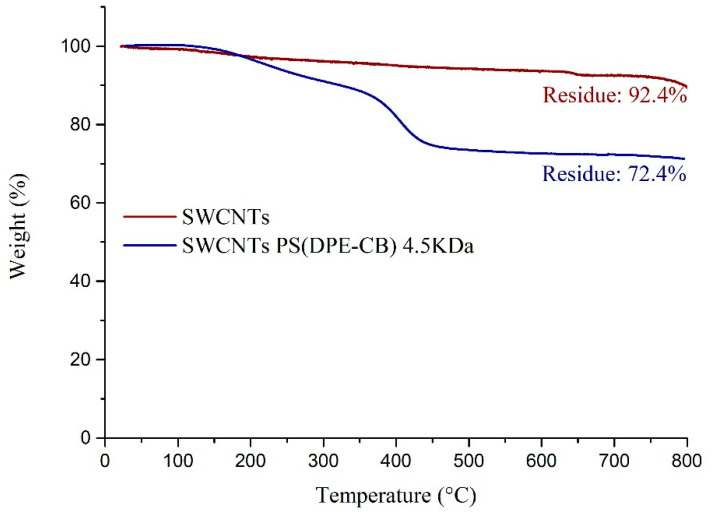
Thermogravimetric analysis under nitrogen atmosphere employing a heating rate of 10 °C/min of pristine SWNTs and SWNTs-*g*-PS.

Our group studied the reaction of several substituted benzocyclobutenes with the *sp*^2^ network of multi-walled carbon nanotubes. We reported that the extent of functionalization can be controlled by the temperature, by the mode of addition of the benzocyclobutene derivative, by the reaction time, and by whether the reaction takes place in bulk vs. solution [34]. For the surface functionalization of the SWNTs with the PS-(DPE-CB), the reaction took place in dibenzyl ether solution at 230 °C for 100 min in total, while the addition of the PS solution to the CNTs dispersion lasted for ten minutes. Thermogravimetric analysis was performed in order to estimate the wt % of grafted polystyrene on the side walls of CNTs and the grafting density of the polymer chain (Figure 4). The grafting density (σ_1_) in mol g^−1^ was calculated based on the %weight of the grafted polymer at *T*_(700°C)_, the molecular weight *M*_n_ of the grafted macromolecule, and by assuming that the remaining samples were constituted purely of carbon. Based on the above procedure, the grafting density (σ_2_) in chains nm^−2^ was calculated according to the polymer chain molecular weight *M*_n_ and the theoretical specific surface of the SWNTs (1315 m^2^ g^−1^) [49]. In summary, the different grafting densities are given by the equations below, σ_1_ = 0.06 mmol g^−1^ and σ_2_ = 0.027 chains nm^−2^.

(1) $$\sigma_{1}=\frac{\%\mathrm{wt}\cdot \mathrm{polymer}}{M_{n}\cdot \%\mathrm{wt}\cdot \mathrm{remaining}\;\mathrm{carbon}\;\mathrm{sample}}\left(\mathrm{mol}/g\right)$$

(2) $$\sigma_{2}=\frac{\sigma_{1}N_{A}}{1315}\left(\mathrm{chain}/m^{2}\right)$$

**Figure 5:**
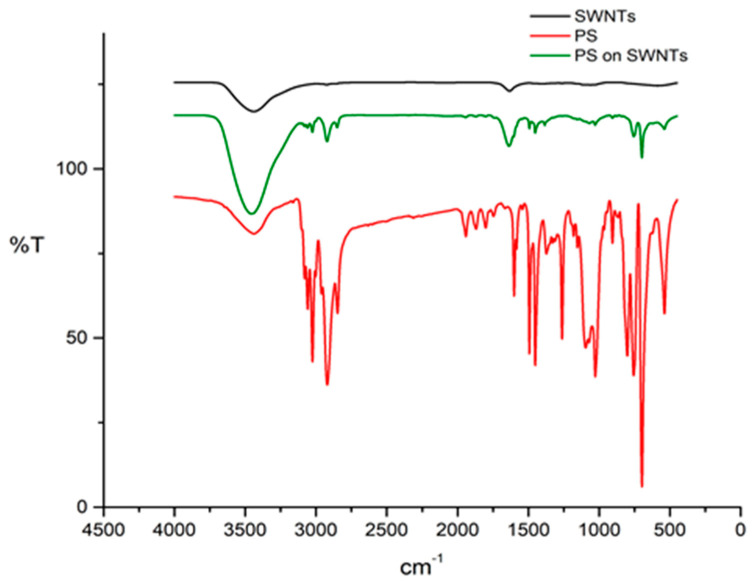
FT-IR spectrum of pristine SWNTs, PS, and PS-*g*-SWNTs.

Adsorption peaks at 3060 and 3026 cm^−1^ due to aromatic C-H stretching vibrations and two peaks at 2928 cm^−1^ and 2858 cm^−1^ corresponding to asymmetric and symmetric stretching of methylene groups –CH_2_ are depicted at the FT-IR spectrum of the PS-*g*-SWNTs in Figure 5. The three characteristic adsorption peaks at 1635, 1492, and 1452 cm^−1^ due to aromatic C=C stretching vibration modes were more obvious compared to the spectrum of pristine SWNTs. Additionally, the peaks at 680–750 cm^−1^ corresponded to the nonaromatic structure of the sample due to the backbone of the PS chain. The peak at 3300 cm^−1^ corresponded to the O-H stretching vibration mode due to the carboxyl groups present at the open tips and at the defect sites at the surface of the CNTs.

**Figure 6:**
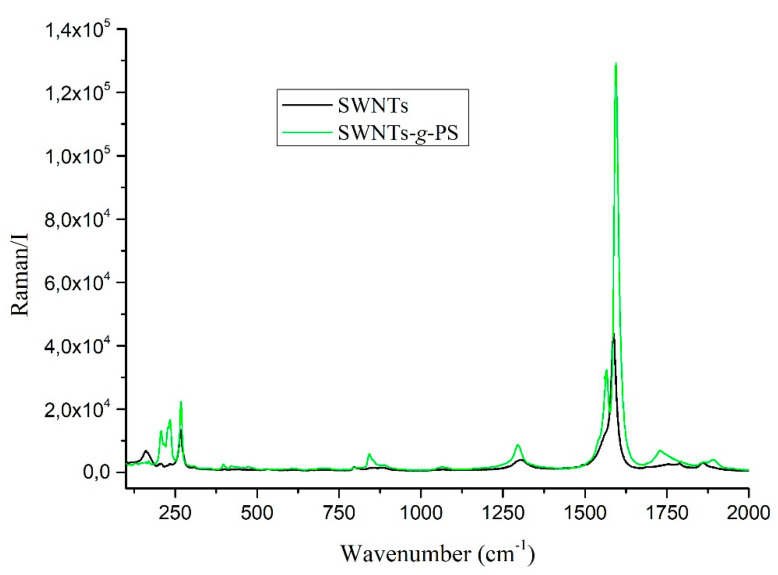
Raman spectra of pristine SWNTs, and SWNTs-*g*-PS 4.5K.

Raman spectroscopy has, for more than two decades, been an exceedingly powerful tool to reveal the exceptional electronic and phonon properties of single-walled carbon nanotubes [50]. The Raman spectrum of SWCNTs mainly consisted of three characteristic peaks. In more detail, the first characteristic (radial breathing mode, RBM) peak was in the area between 100 and 400 cm^−1^ and was related to the tube diameter and length. A second peak between 1500 and 1600 cm^−1^ was related to the tangential G-mode of graphite, which occurred at 1582 cm^−1^. In SWNTs, this peak originated from breaking the symmetry of the tangential vibration when the graphene sheet was rolled into a tube consisting of two components. One located at around 1590 cm^−1^ (G^+^) was due to vibration of carbon atoms along the nanotube axis and was sensitive to charge transfer from dopant additions to SWNTs. Another at around 1570 cm^−1^ (G^−^) was assigned to a vibration of carbon atoms along the circumferential direction of SWNTs. The last characteristic peak around 1350 cm^−1^, the disorder-induced D-mode, was attributed to a double resonant Raman scattering process involving a defect or disorder in CNTs, such as amorphous carbon, vacancies, and heteroatoms [51]. The RBMs showed changes after grafting of PS where more peaks were present in the area between 100–400 cm^−1^ after the functionalization reaction. These peaks were attributed to metallic and semiconducting SWNTs, and the presence of more peaks was indirect evidence of CNTs debundling due to polymer functionalization, as reported elsewhere [52]. The structural integrity of the surface grafted MWNTs was confirmed by Raman spectroscopy, which showed both the disorder (D) and the tangential (T) bands at 1334 and 1590 cm^−1^, respectively. A slight increase in the peak ratio of *I*_D_/*I*_T_ = 0.16 of the functionalized as compared to the pristine SWNTs (*I*_D_/*I*_T_ = 0.13) was attributed to enhanced attachment of PS chains through the Diels–Alder thermal cycloaddition (Figure 6). The increase in the disorder mode band corresponded to the conversion of the hybridization of the C atoms on the nanotubes from *sp*^2^ to *sp*^3^, which indicated covalent side-wall functionalization.

**Figure 7:**
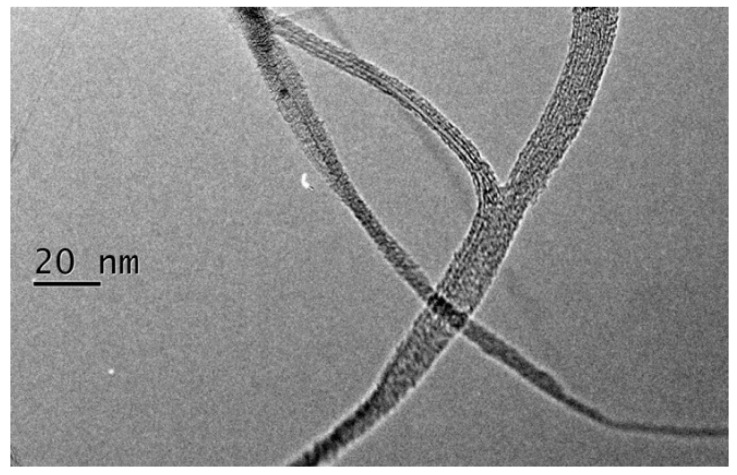

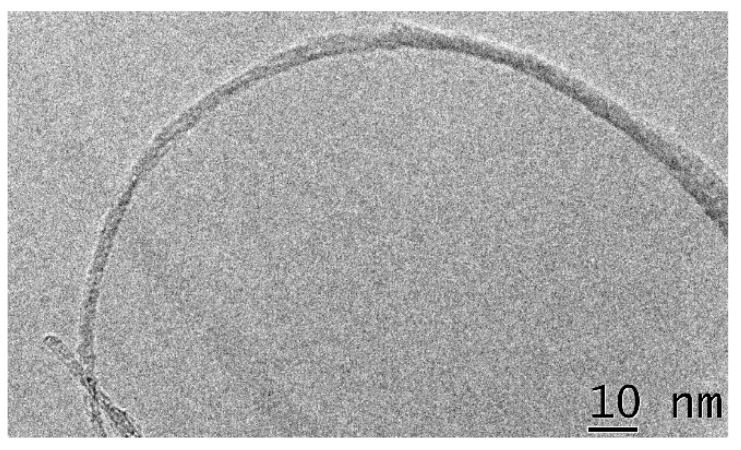
TEM images of SWNTs-*g*-PS.

**Figure 8:**
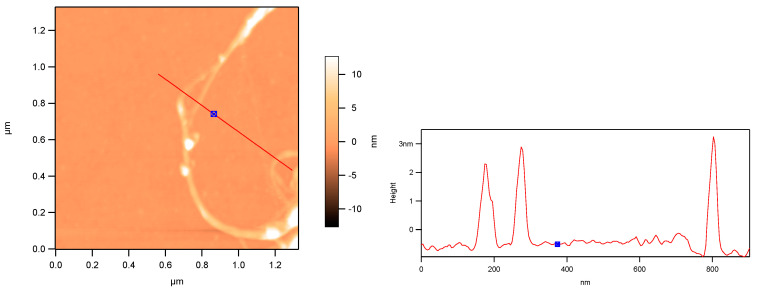
Atomic force microscopy (AFM) image of SWNTs-*g*-PS.

The direct evidence for the presence of grafted PS on the SWNTs was obtained from TEM and AFM images, as it can be seen in Figure 7 and Figure 8, respectively. A dilute solution of SWNTs-*g*-PS in chloroform was drop-coated onto a carbon coated copper grid, and the thin film was visualized with TEM. A typical TEM image of SWNTs-*g*-PS showed amorphous coating on the surface of SWNTs in selected regions (Figure 7). Certain areas of the SWNT surfaces were observed to have no polymer coverage, indicating a non-uniformity of the polymer grafting. The surface of SWNTs was covered with the PS layer inhomogeneously, which suggested a non-uniform grafting of PS chains around the surface of carbon nanotubes.

Atomic force microscopy was performed to visualize the grafted polystyrene chains on the surface of SWNTs. Usually, large CNT bundles were observed before functionalization, however, the PS functionalized SWNTs existed in small aggregates, and even individual SWNTs were observed by AFM. The average height of the pristine SWNTs ranged from 0.8 to 1.5 nm. As shown in Figure 8, the height profile demonstrated the existence of an individual single walled carbon nanotube. It is also clear from the figure that the nanotubes maintained their length (>1 μm), since they did not suffer any surface pre-modification reaction prior to the “grafting to” functionalization. An increase up to ~3 nm of the height profiles of the AFM images taken after the surface functionalization of SWNTs with the polystyrene polymer chains was revealed. This increase could be attributed either to enhanced polymer grafting efficiency or to the existence of small aggregates.

**Figure 9:**
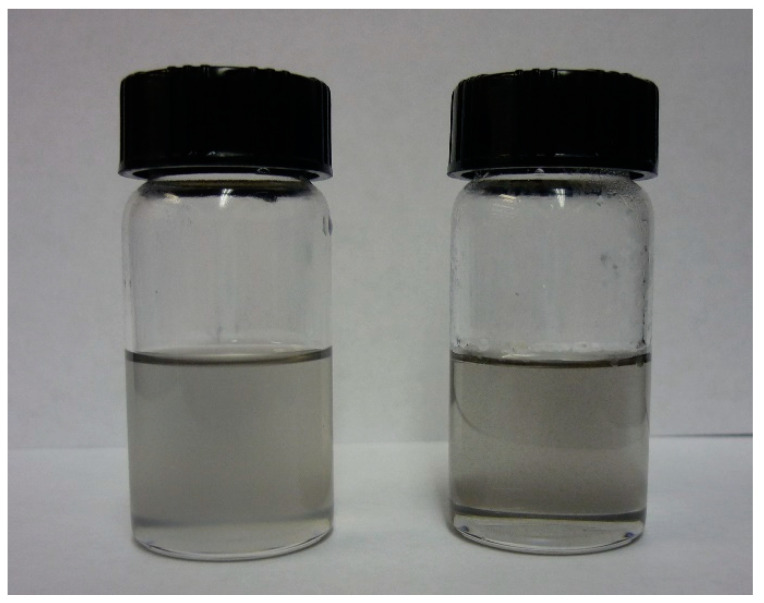
Dispersion of pristine SWNTs and SWNTs-*g*-PS in THF (left photo) and in CHCl_3_ (right photo) at room temperature (0.3 mg mL^−1^).

The SWNTs-*g*-PS exhibited good solubility in common PS organic solvents such as tetrahydrofuran and chloroform, where no precipitation appeared from the homogeneous dispersion even after one week at room temperature (Figure 9).

## 4. Conclusions

A facile, one-step [4+2] Diels–Alder thermal cycloaddition reaction for the surface modification of single-walled carbon nanotubes without compromising their structural integrity was performed. A well-defined polystyrene with a diphenylethylene cyclobutene terminal group was synthesized by anionic polymerization and grafted on the CNT side walls in a dibenzyl ether solution at 230 °C in the absence of any catalyst and without any previous modification of the SWNT surface. The quantitative end group functionalization of the polystyrene chains was confirmed by MALDI TOF mass and ^1^H NMR spectroscopies. TEM and AFM microscopy revealed the presence of an inhomogeneous thin film on the CNT surface. The grafting density was also estimated by thermogravimetric analysis, and the SWNTs-*g*-PS were well dispersed in organic solvents.

## Data Availability

The data presented in this study are available in this manuscript and the Supplementary Information Section.

## Supplementary Materials

The following are available online at https://www.mdpi.com/article/10.3390/polym13071169/s1, Figure S1. ^1^H-NMR spectrum of DPE-CB.
