# Supplementary material for: Functionalization of Single-Walled Carbon Nanotubes with End-Capped Polystyrene via a Single-Step Diels–Alder Cycloaddition
Source: Polymers (Basel). 2021 Apr 6;13(7):1169. doi: 10.3390/polym13071169 (PMC8038725; doi:10.3390/polym13071169)
Supplement: Supplementary file 1 [file polymers-13-01169-s001.pdf]

Functionalization of Single-Walled Carbon Nanotubes with End-Capped Polystyrene *via* a Single-Step Diels-Alder Cycloaddition

Maria-Malvina Sathouraki,[1] Christos Pantazidis,[1] Emmanouil Mygiakis,[1] Apostolos Avgeropoulos,[2] and Georgios Sakellariou[1*]

[1]Department of Chemistry, National and Kapodistrian University of Athens, Panepistimiopolis Zografou, 15771 Athens, Greece

[2]Department of Materials Science and Engineering, University of Ioannina, 45110 Ioannina, Greece

E-mail: gsakellariou@chem.uoa.gr

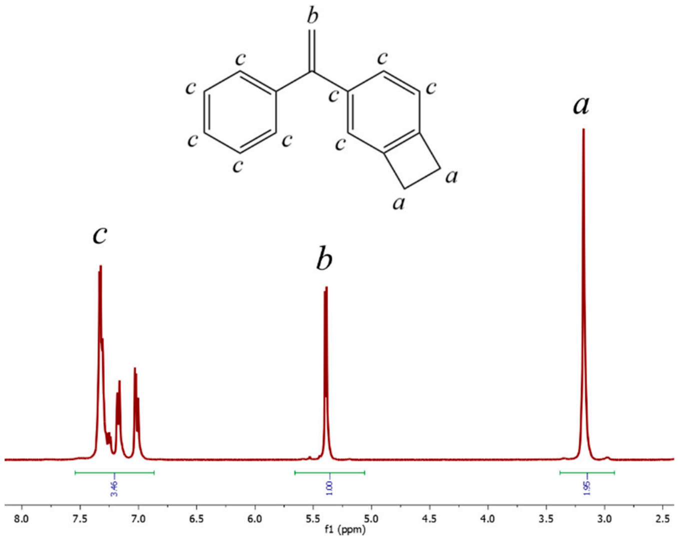

Figure S1. ^{1}H-NMR spectrum of DPE-CB.

^{1}H-NMR (400 MHz, $CDCl_3$) δ: 7.27 (d, 1H, ArH), 7.2 (s, 1H, ArH), 7.05 (d, 1H, ArH), 6.73 (dd, 1H, CH), 5.7 (d, 1H, CH_2), 5.2 (d, 1H, CH_2), 3.22 (s, 4H, CH_2).
